# MULTIDISCIPLINARY COLLABORATION AS A IMPROVEMENT IN PROVIDING QUALITY USER-ORIENTED SERVICES

**DOI:** 10.1192/j.eurpsy.2023.1906

**Published:** 2023-07-19

**Authors:** G. Racetovic, M. Latinovic, S. Grujic-Timarac

**Affiliations:** 1 Health Center Prijedor, Community Mental Health Center Prijedor, Prijedor; 2 Depity of Minister of Health, Ministry of Health and Social Welfare of the Republic of Srpska, Banja Luka; 3 Department for Psychiatry, Hospital “Dr Mladen Stojanovic” Prijedor, Prijedor, Bosnia and Herzegovina

## Abstract

**Introduction:**

As one of main aim of broad mental health (MH) reform in Bosnia and Herzegovina (BH), quality of services and continuous improvement are priorities. In last decade network of community services-community mental health centers (CMHC) covered all the parts of the country and is connected with secondary and tertiary medical institutions as well as with broad network of centers for social welfare and growing number of user organizations.

**Objectives:**

As a novel practice that will be involved in standards of quality for all MH services is joint planning of hospital discharge people with mental illnesses, supported by legislative and connected with other positive results of mental health reform in BH

**Methods:**

Review of implementation of results of MH reform in BH with focus on joint planning hospital discharge.

**Results:**

In BH in last decade has been established broad spectrum of services that improved quality of care through multidisciplinary collaboration between sectors. Important role has given to users’ organization. Quality standards are defined trough certification and accreditation. New services were developed or renewed and implemented (such as case management, occupational therapy). Entities’ policies and strategies involved new services and improved MH legislative following the course of more involvement of patients as well as their families, representatives or persons of their trust in decision making and planning of multidisciplinary treatments in the CMHCs. Joint Planning of discharge from the hospital fund as important step in further improvement of quality of care for people with MH disorders, especially for those with severe mental illnesses.

**Conclusions:**

Last decade in BH gave important results in the better quality of MH care. Further plans will be focused on implementation of new MH user-oriented services.

**Disclosure of Interest:**

None Declared

